# A Novel Time-Dependent CENP-E Inhibitor with Potent Antitumor Activity

**DOI:** 10.1371/journal.pone.0144675

**Published:** 2015-12-09

**Authors:** Akihiro Ohashi, Momoko Ohori, Kenichi Iwai, Tadahiro Nambu, Maki Miyamoto, Tomohiro Kawamoto, Masanori Okaniwa

**Affiliations:** 1 Oncology Drug Discovery Unit, Pharmaceutical Research Division, Takeda Pharmaceutical Company Limited, Fujisawa, Japan; 2 DMPK Research Laboratories, Pharmaceutical Research Division, Takeda Pharmaceutical Company Limited, Fujisawa, Japan; 3 Biomolecular Research Laboratories, Pharmaceutical Research Division, Takeda Pharmaceutical Company Limited, Fujisawa, Japan; Virginia Tech, UNITED STATES

## Abstract

Centromere-associated protein E (CENP-E) regulates both chromosome congression and the spindle assembly checkpoint (SAC) during mitosis. The loss of CENP-E function causes chromosome misalignment, leading to SAC activation and apoptosis during prolonged mitotic arrest. Here, we describe the biological and antiproliferative activities of a novel small-molecule inhibitor of CENP-E, Compound-A (Cmpd-A). Cmpd-A inhibits the ATPase activity of the CENP-E motor domain, acting as a time-dependent inhibitor with an ATP-competitive-like behavior. Cmpd-A causes chromosome misalignment on the metaphase plate, leading to prolonged mitotic arrest. Treatment with Cmpd-A induces antiproliferation in multiple cancer cell lines. Furthermore, Cmpd-A exhibits antitumor activity in a nude mouse xenograft model, and this antitumor activity is accompanied by the elevation of phosphohistone H3 levels in tumors. These findings demonstrate the potency of the CENP-E inhibitor Cmpd-A and its potential as an anticancer therapeutic agent.

## Introduction

Antimitotic drugs targeting microtubule dynamics, such as taxanes and vinca alkaloids, are widely used in the clinical treatment of cancer [1]. However, peripheral neuropathy is a major adverse effect of these drugs, presumably because they directly inhibit the assembly of microtubule structures even in non-dividing neural cells [2]. To reduce the incidence of this debilitating side effect, the components of mitotic spindles that are non-structural but essential for mitosis have recently attracted attention as target molecules for next-generation anticancer drugs. Two mitotic kinesins, Eg5 (also called kinesin spindle protein; KSP) and centromere-associated protein E (CENP-E), are also emerging as promising target molecules for anticancer drugs [3]. Although CENP-E and Eg5 are both mitotic spindle motor proteins of the kinesin superfamily [4], their molecular regulatory functions are distinct. Eg5 regulates centrosome separation and bipolar mitotic spindle formation [5–7], whereas CENP-E is localized at the kinetochore of chromosomes [4, 8] and controls chromosome alignment during metaphase by capturing the microtubule plus end at the kinetochore [9–11]. More recently, CENP-E has been reported to transport the pole-proximal chromosomes toward the metaphase plate, and CENP-E-driven chromosome congression is guided by tubulin post-translational modification [12].

The most advanced mitotic kinesin inhibitor is an Eg5 inhibitor, ispinesib, which has progressed to Phase II clinical trials [6, 13–15]. A number of other small-molecule Eg5 inhibitors have been or are being evaluated in clinical trials, including AZD4877, ARRY-520, SB-743921, ARQ-621, LY2523355, MK-0731, and EMD-534085 [16–23]. In contrast, to date, only one small-molecule CENP-E inhibitor, GSK923295, has been evaluated in clinical trials [24–26]. GSK923295 is an allosteric small-molecule inhibitor that targets CENP-E motor activity and exhibits potent antitumor activity in the preclinical models of various human tumor xenografts [24, 27, 28]. Although CENP-E has the potential as a target molecule for anticancer drugs, the limited number of CENP-E inhibitors undergoing clinical trials or even preclinical studies possibly reflects the difficulties involved in the development of potent and selective inhibitors of CENP-E with adequate pharmaceutical potency.

We have developed a novel time-dependent CENP-E inhibitor, Compound-A (Cmpd-A), based on a biochemical screening of the ATPase activity of the CENP-E motor domain [29]. Here, we report the characterization of Cmpd-A on the enzymatic mode of action, cellular morphology, pharmacokinetics (PK), and pharmacodynamics (PD) and demonstrate its antiproliferative activities both *in vitro* and *in vivo*. Our investigations contribute to an increased understanding of the linking chromosome instability and antiproliferative activity in cancer cells and confirm that small-molecule inhibitors of CENP-E motor activity have important potential as anticancer drugs.

## Materials and Methods

### Compounds

(+)-*N*-[7-Cyano-1,1-dioxido-6-(trifluoromethyl)-2,3-dihydro-1-benzothiophen-3-yl]-*N*-[2-(dimethylamino)ethyl]-3-(4-fluoro-3-methylphenyl)-5-methoxyimidazo[1,2-*a*]pyridine-2-carboxamide (Cmpd-A) was synthesized by Takeda Pharmaceutical Company Ltd [29].

### CENP-E enzyme assay

An ATPase assay was used to determine human CENP-E activity. The CENP-E motor domain was purchased from Cytoskeleton, Inc. (Denver, CO, USA). The ATPase assay was performed using 62.5 ng/mL of the CENP-E motor domain, 22 μg/mL of microtubules (Cytoskeleton Inc.), and 1.25 μM or 500 μM of ATP. Reactions were performed in 6 μL of reaction buffer [20 mM piperazine-*N*,*N*′-bis(2-ethanesulfonic acid) (PIPES)-KOH, pH 6.8, 3.0 mM MgCl_2_, 3.0 mM KCl, 1.0 mM ethylene glycol tetraacetic acid (EGTA), 1.0 mM dithiothreitol, 0.01% (w/v) Brij-35, and 0.2% (w/v) bovine serum albumin] for 60 min at room temperature. The amount of ADP produced during the ATPase reaction was determined with an ADP-Glo kit (Promega, Madison, WI, USA). The luminescence was measured using an Envision plate reader (PerkinElmer, Inc., Waltham, MA, USA).

### Cell cultures

HeLa, DU145, COLO205, NIH-OVCAR3, RKO, ES2, SK-OV3, PC-3, SW620, SW480, CAPAN-2, Panc 04.03, and MRC5 cell lines were purchased from American Type Culture Collection (Manassas, VA, USA). TCC-PAN2, TYK-nu, and OVTOKO cell lines were purchased from the Japanese Collection of Research Bioresources Cell Bank (Osaka, Japan). HeLa cells were cultured in Dulbecco’s Modified Eagle’s Medium supplemented with 10% fetal bovine serum (FBS). DU145, TYK-nu, and MRC5 cells were cultured in Modified Eagle’s Medium supplemented with 10% FBS. COLO205, RKO, NIH-OVCAR3, TCC-PAN2, OVTOKO, and Panc 04.03 cells were cultured in RPMI 1640 medium supplemented with 10% FBS. PC-3 cells were cultured in Ham’s F12 nutrient mixture supplemented with 10% FBS. SKOV3, ES2, and CAPAN-2 cells were cultured in McCoy’s 5a medium supplemented with 10% FBS. SW620 and SW480 cells were cultured in Leibovitz’s L-15 medium supplemented with 10% FBS.

### Cell growth and caspase-3/7 assays

Cell growth assays were performed as described previously [29]. Cell growth was evaluated based on intracellular ATP concentrations using a CellTiter-Glo luminescent cell viability kit (Promega Corp.. Caspase-3/7 activity was evaluated using a caspase-3/7-Glo luminescent kit (Promega). Chemiluminescence was measured with a microplate reader.

### Cell cycle synchronization

To synchronize cells at the G1/S phase, a double thymidine (dT) block was prepared. Cells were treated with 2 mM thymidine (Sigma-Aldrich, St. Louis, MO, USA) for 16 h (first block), followed by incubation in thymidine-free medium for 8 h. The cells were then treated again with 2 mM thymidine for 16 h (second block). The cells were collected at 0, 2, 4, 6, 8, 10, and 12 h after release from the second block.

### Immunofluorescence

Immunofluorescence was performed as described previously [30] using the following antibodies: anti-CENP-B (sc22788; Santa Cruz Biotechnology, Santa Cruz, CA, USA), anti-HEC1 (ab3613; Abcam, Cambridge, UK), anti-BubR1 (612503; BD Transduction Laboratories, San Jose, CA, USA), and anti-α-tubulin (T9026; Sigma-Aldrich). Images were captured with a Plan-APOCHROMAT 100× oil lens on an Axiovert 200M microscope (Carl Zeiss, Jena, Germany).

### Immunoblotting

Immunoblotting was performed as described previously [30]. The following antibodies were used at a concentration of 0.1–0.5 μg/mL: anti-BubR1 (1:1,000 dilution; 612503; MD Transduction), anti-pHH3 (1:1,000 dilution; 06570; Upstate Biotechnology), anti-Cyclin B1 (1:1,000 dilution; sc752; Santa Cruz Biotechnology), and anti-GAPDH (1:10,000 dilution; MAB374; Chemicon). Immunoblotted proteins were visualized by chemiluminescence.

### Transfection of small-interfering RNA (siRNA) oligonucleotides

Pools of four (SMART pools, Dharmacon, Lafayette, CO, USA) predesigned siRNA oligonucleotides per gene of interest were tested. siRNA oligonucleotides targeting BubR1 were obtained from Dharmacon (D-004101). The siTrio negative control (B-Bridge International, Inc., Mountain View, CA, USA) was used as a non-silencing (NS) siRNA (siNS). Fifty nanomoles of pooled siRNA per gene were used. siRNA transfection was performed as described previously [30]. Transfection of siRNA oligonucleotides was performed with Dharmafect (Dharmacon) in 6-well plates according to the manufacturer’s specifications.

### RNA preparation and TaqMan quantitative Real Time-polymerase chain reaction (RT-PCR) analysis

Total RNA was extracted using the RNeasy Miniprep kit (Qiagen). cDNAs were synthesized from 500 ng of total RNA using the TaqMan Reverse Transcription Reagent kit (Applied Biosystems, Foster City, CA, USA). RT-PCR was performed using an ABI PRISM 7900 instrument according to the manufacturer’s protocol (Applied Biosystems). The 6-carboxyfluorescein fluorescence emitted from each sample was measured as a function of the PCR cycle number (Ct) using the ABI PRISM 7900 instrument. The gene expression was calculated by the comparative Ct method [31]. The expression ratios of the indicated genes were quantified using the GAPDH expression in each cell line as a control.

### Immunohistochemistry

Immunohistochemistry was performed as described previously [29]. Briefly, endogenous peroxidases were quenched by the addition of 3% H_2_O_2_. Antigen retrieval was performed by heating the samples in 10 mM citrate buffer (pH 6.0). The sections were incubated with a rabbit anti-phosphohistone H3 (pHH3) antibody (#06–570, Millipore, Billerica, MA, USA). The peroxidase-conjugated secondary antibody (Histofine Simple Stain Mouse MAX PO; Nichirei Bioscience, Tokyo, Japan) was used for color development with diaminobenzidine tetrahydrochloride.

### *In vivo* PK/PD studies

Colo205 was xenografted into 5-week-old nude mice by subcutaneous injection (5 × 10^6^ cells/mouse). Cmpd-A, dissolved in 0.1 M citric acid with 10% dimethyl sulfoxide (DMSO), 9% Cremophor EL, and 18% PEG 400, was intraperitoneally administered into xenografted mice with a tumor volume of 150–400 mm^3^ at a dose of 100 mg/kg. After a single administration, tumors and plasma were collected at the indicated time points for the measurement of drug concentrations (PK) by liquid chromatography-tandem mass spectrometry. The pHH3 levels in the tumor at the same time points were also detected by immunoblotting (PD).

### *In vivo* efficacy studies

*In vivo* efficacy studies were performed as described previously [29]. COLO205 cells (3–5 × 10^6^ cells/mouse) were xenografted into nude mice. Mice bearing tumors (100–250 mm^3^) were selected and randomly categorized into vehicle and Cmpd-A groups (five mice/group). The xenografted mice were intraperitoneally administrated with 100 mg/kg Cmpd-A or vehicle three times (0, 8, and 24 h) on the first day of the study. The antitumor activity (T/C %) was calculated using the following formula:

$$\text{antitumor activity}\;(\text{T}/\text{C}\;\%)=[\frac{\left(Cmpd–A\;tumor\;volume–Cmpd–A\;tumor\;volume\;on\;day\;0\right)}{\left(vehicle\;tumor\;volume–vehicle\;tumor\;volume\;on\;day\;0\right)}]\times 100$$

All *in vivo* procedures were performed in accordance with protocols approved by the Institutional Animal Care and Use Committee at Takeda Pharmaceutical Co. Ltd. (Experimental Protocol Number: 00004407).

### Fluorescence-activated cell sorting (FACS) analysis

FACS analysis was performed as described previously [29]. Briefly, cells fixed with 70% ethanol were incubated for 30 min in phosphate-buffered saline containing 2% FBS, 100 μg/mL RNase A (Sigma-Aldrich), and 1 μg/mL Alexa Fluor 488-conjugated pHH3 antibodies (Cell Signaling Technology, Danvers, MA, USA). After washing, the cells were incubated in 50 μg/mL propidium iodide (Sigma-Aldrich). In total, 10,000 cells were analyzed by FACSCalibur (Becton Dickson, Franklin Lakes, NJ, USA).

## Results

### Cmpd-A is a time-dependent CENP-E inhibitor that causes chromosome misalignment during mitosis

**Fig 1:**
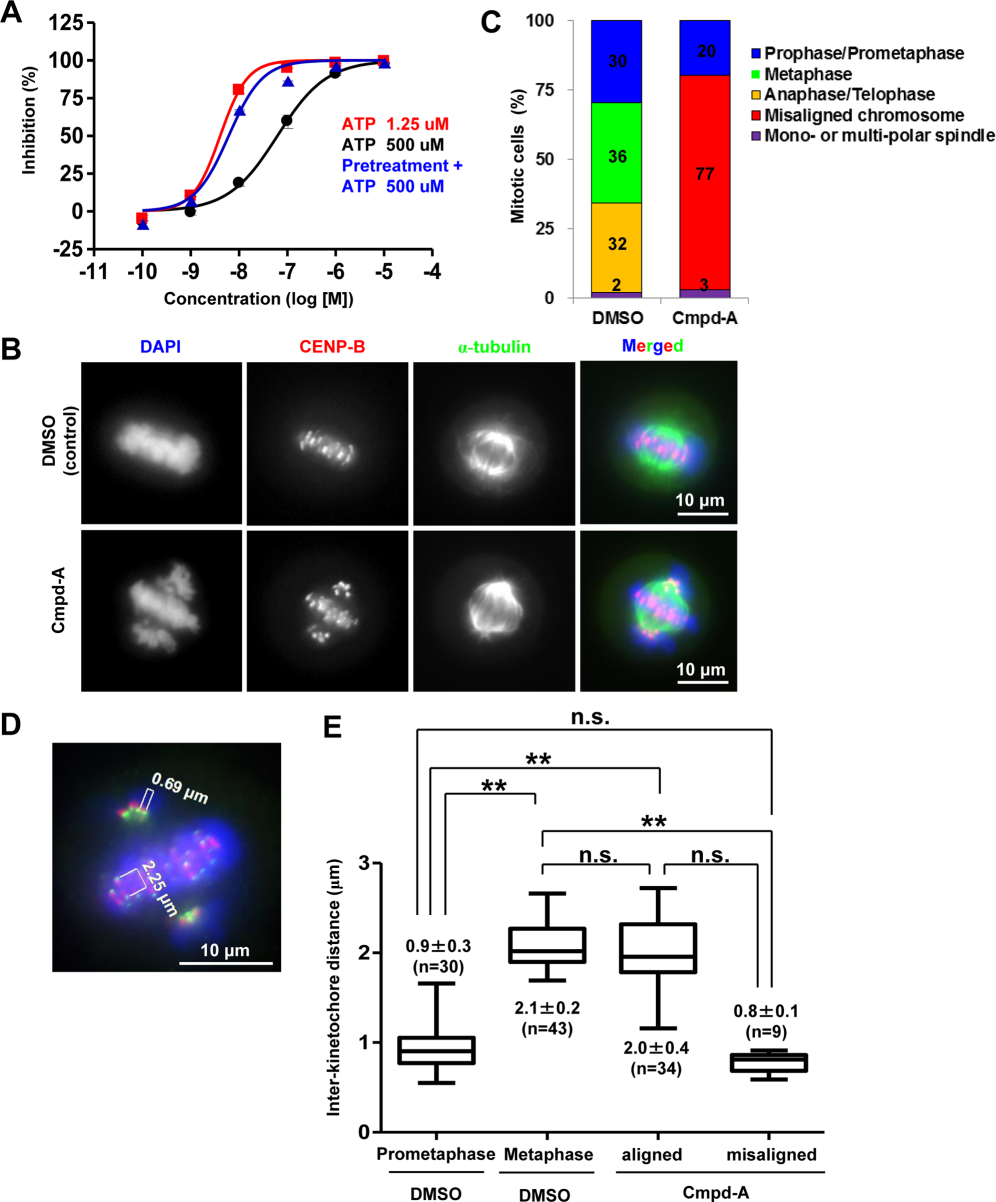
CENP-E inhibitor Cmpd-A induces chromosome misalignment during mitosis. (A) Cmpd-A is a time-dependent inhibitor with an ATP competitive-like behavior. Red and black lines indicate the dose-dependent activity of Cmpd-A in the presence of low (1.25 μM) and high (500 μM) concentrations of ATP, respectively. The blue line indicates the activity of Cmpd-A with a high concentration of ATP, following 1 h of preincubation with CENP-E. The X-axis and Y-axis indicate the concentration of Cmpd-A and % inhibition of CENP-E ATPase activity, respectively. (B) Representative mitotic HeLa cells treated with Cmpd-A (200 nM) or DMSO. Arrows indicate misaligned chromosomes. Blue, green, and red signals indicate 4′,6-diamidino-2-phenylindole (DAPI)-stained DNA, α-tubulin, and CENP-B (kinetochores), respectively. (C) Quantitative analysis of mitotic morphology in the DMSO- or Cmpd-A-treated HeLa cells. The cells were treated for 3 h with 200 nM Cmpd-A or DMSO after dT block release. The DMSO- and Cmpd-A-treated mitotic cells (105 and 106 cells, respectively) were then counted. (D) Inter-kinetochore distance of aligned and misaligned chromosomes in HeLa cells treated with Cmpd-A or DMSO. Prometaphase (left) and metaphase (middle) cells were used as controls for misaligned and aligned chromosomes, respectively. The inter-kinetochore distance was measured between the outer kinetochore markers (HEC1) of individual chromosomes. Statistical analysis was performed using Student’s t-test. Differences were considered significant at P ≤ 0.05 (*) and P ≤ 0.01 (**).

Cmpd-A was developed as a small-molecule inhibitor of CENP-E [29]. Cmpd-A inhibits the ATPase activity of the CENP-E motor domain but not that of the other kinesins, such as Eg5 and kinesin-1 heavy chain [29]. While the binding site of CENP-E with our imidazo[1,2-*a*]pyridine class CENP-E inhibitor has not been not fully identified, our previous studies using site-directed mutagenesis revealed that Cmpd-A binds to the allosteric site of CENP-E formed by the L5 loop juxtaposed to the ATP-binding site [32][33]. Enzymatic assays, however, revealed that the activity of Cmpd-A was limited at high ATP concentrations (Fig 1A, black line), suggesting that this molecule potentially competes with ATP for its binding. However, its inhibitory activity was sustained even at high ATP concentrations following 1-h preincubation with the CENP-E motor domain (Fig 1A, blue line). The results of this investigation indicated that Cmpd-A is a time-dependent inhibitor that has the potential to overcome high ATP concentrations in cellular settings when it occupies the allosteric site of CENP-E.

Next, the cellular effects of Cmpd-A on chromosome dynamics during mitosis were assessed in HeLa cells. Using its motor activity, CENP-E captures microtubules on kinetochores to control chromosome alignment at the metaphase plate. Therefore, the enzymatic inhibition of CENP-E motor activity by Cmpd-A is also expected to induce chromosome misalignment. Synchronous cells were used to monitor the cell cycle-dependent effects of Cmpd-A in detail. HeLa cells were synchronized at the G1/S phase by a dT block, and 7 h after release from the dT block, which corresponds to the G2 phase, the cells were treated for 3 h with 200 nM Cmpd-A or dimethyl sulphoxide (DMSO) as a negative control. Immunofluorescence of α-tubulin and CENP-B, which are markers of mitotic spindles and kinetochores, respectively, was conducted to observe chromosome alignment on the metaphase plate in HeLa cells. Although chromosomes were aligned at the metaphase plate in the mitotic HeLa cells treated with DMSO (Fig 1B, upper panels), treatment with Cmpd-A caused chromosome misalignment (Fig 1B, lower panels) despite the formation of bipolar mitotic spindles (Fig 1B, lower panels, α-tubulin). Quantitative analysis demonstrated that 77% of mitotic cells treated with Cmpd-A exhibited pole-proximal chromosome misalignment, while DMSO-treated cells did not (Fig 1C). On the contrary, metaphase cells with aligned chromosomes (36%) and anaphase/telophase cells (32%) were observed in DMSO-treated cells, but not in the Cmpd-A-treated cells. These results indicate that CENP-E inhibition by Cmpd-A induces pole-proximal chromosome misalignment, and cell cycle transition to anaphase/telophase appears to be inhibited in these cells.

The inter-kinetochore distance between the outer kinetochores was measured with an outer kinetochore marker HEC1 flanking the inner kinetochore marker CENP-B (Fig 1D). In the DMSO-treated cells, the inter-kinetochore distances of the aligned chromosomes at metaphase were significantly longer than those of the misaligned chromosome at prometaphase (Fig 1E). On the contrary, the inter-kinetochore distances of the misaligned chromosomes in the Cmpd-A-treated cells were significantly reduced compared with those of the aligned chromosomes in the DMSO- and Cmpd-A-treated cells (Fig 1E), while the inter-kinetochore distances of the aligned chromosomes between the Cmpd-A- and DMSO-treated cells were not significantly different. Given that CENP-E guides pole-proximal chromosomes toward the equatorial plate [12], the inhibition of CENP-E motor activity by Cmpd-A appears to induce chromosome misalignment of pole-proximal chromosomes. Because pole-proximal misaligned chromosomes are expected to possess syntelic or monotelic attachments, the inter-kinetochore tension of these sister chromatids would be weakened during formation of the mitotic spindle.

**Fig 2:**
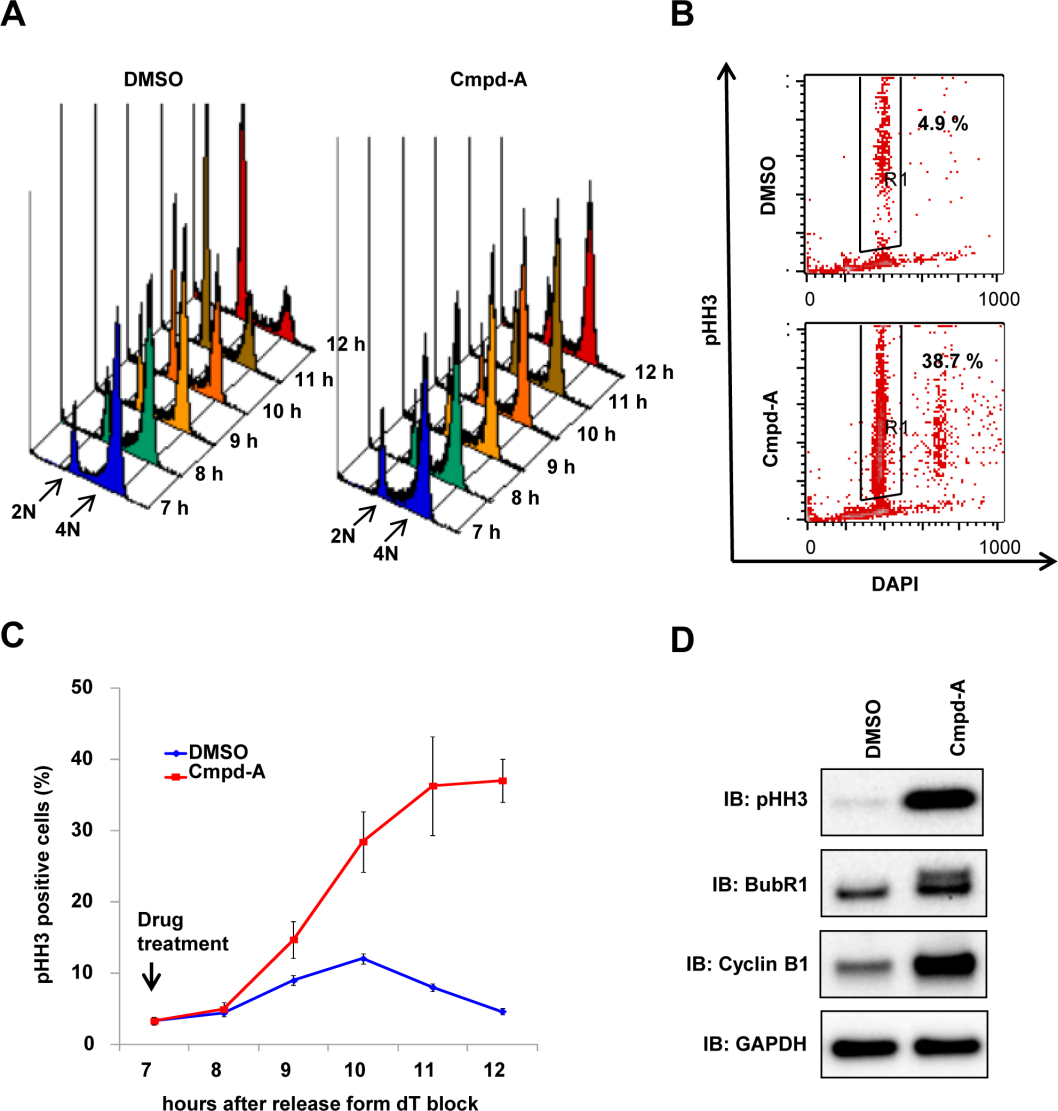
Cmpd-A induces prolonged mitotic arrest accompanied by SAC activation. (A) Cell cycle histogram of synchronous HeLa cells treated with Cmpd-A (200 nM) or DMSO. Cmpd-A was added at the G2 phase (7 h after dT block release), and the cells were collected at the indicated time points for FACS analysis. (B) pHH3 in synchronous HeLa cells treated with Cmpd-A (200 nM) or DMSO. Cmpd-A was added at the G2 phase (7 h after dT block release), and the cells were collected 12 h after dT block release. Representative results are shown. (C) pHH3 elevation in synchronous HeLa cells treated with Cmpd-A (200 nM) or DMSO. Cmpd-A was added at the G2 phase (7 h after dT block release), and the cells were collected at the indicated time points for FACS analysis of pHH3 staining. The graph indicates quantified pHH3-positive cells (mean ± standard deviation; n = 3). Red and blue lines indicate Cmpd-A- and DMSO-treated HeLa cells, respectively. (D) Immunoblotting of mitosis markers in synchronous HeLa cells treated with Cmpd-A or DMSO. The cells were treated with Cmpd-A or DMSO as described in Fig 2A and collected 12 h after dT block release for immunoblotting.

The spindle assembly checkpoint (SAC) machinery detects the aberrant attachment between microtubules and kinetochores and blocks the onset of anaphase until the kinetochores of each duplicated chromosome pair have achieved a bipolar attachment to the mitotic spindle [34–36]. The next step was to determine whether chromosome misalignment by Cmpd-A causes SAC-mediated mitotic arrest. To monitor the cell cycle-dependent effects of Cmpd-A in detail, synchronous cells were used for time-course cell cycle analysis. Cell cycle progression from the G2 phase through mitosis to the next G1 phase was followed in HeLa cells either treated or not treated (DMSO controls) with 200 nM of Cmpd-A 7 h after dT block release. The control cells went through the G2/M phase (4N) into the next G1 phase (2N) within 11 h of dT block release (Fig 2A, left), whereas the cells treated with Cmpd-A were stalled at the G2/M phase (4N; Fig 2A, right). Furthermore, the time-dependent accumulation of pHH3-positive cells (i.e., mitotic cells) was observed in the Cmpd-A-treated cells (Fig 2B and 2C). BubR1 phosphorylation and cyclin B1 accumulation, both of which indicate SAC activation, were also detected in Cmpd-A-treated cells (Fig 2D). These results demonstrate that chromosome misalignment induced by Cmpd-A activates the SAC machinery to induce prolonged mitotic arrest.

### Cmpd-A activates SAC leading to antiproliferation

**Fig 3:**
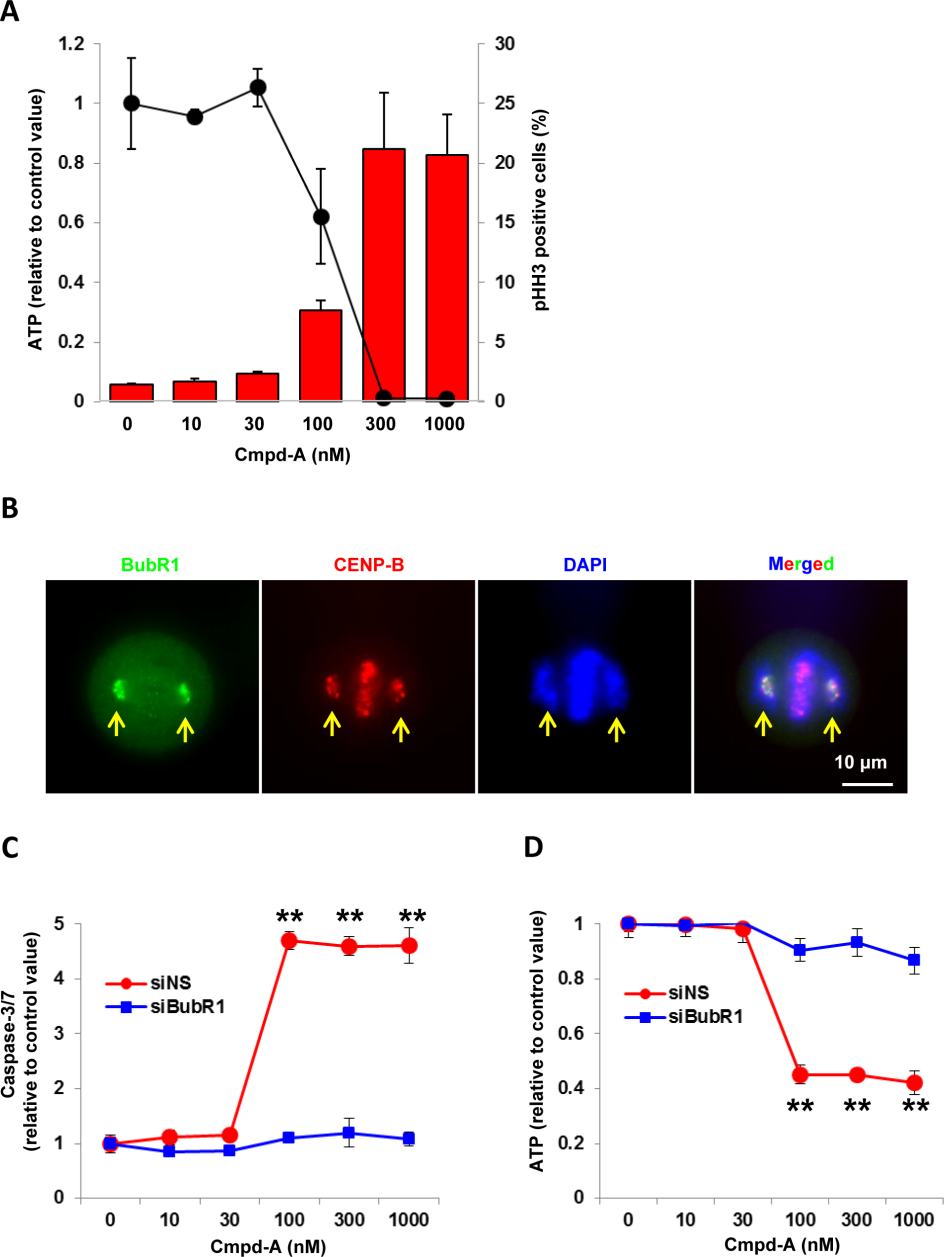
Cmpd-A exhibits potent antiproliferative activity in HeLa cells accompanied by the accumulation of pHH3. (A) Correlation between antiproliferative activity and pHH3 elevation in Cmpd-A-treated HeLa cells. The line graph and red bars indicate the relative ATP amount and pHH3-positive cells (%), respectively. Data are presented as mean ± standard deviation (SD) (n = 3). HeLa cells were treated with Cmpd-A at the indicated concentrations. The cells were collected at 72 h and 24 h after treatment for cell viability and FACS analysis, respectively. The relative ATP amount was calculated based on chemiluminescence compared with the 0 nM chemiluminescence value (control). (B) Representative immunofluorescence of BubR1 in mitotic HeLa cells treated with Cmpd-A (200 nM). Arrows indicate misaligned chromosomes. Green, red, and blue signals indicate BubR1, CENP-B, and DAPI-stained DNA, respectively. (C) BubR1-dependent caspase-3/7 activation in response to Cmpd-A treatment. Twenty-four hours after siNS or siBubR1 treatment, the cells were treated with Cmpd-A at the indicated concentration for 24 h. Relative caspase-3/7 activities were calculated based on chemiluminescence compared with the 0 nM chemiluminescence value in each siRNA treatment. Statistical analysis was performed using Student’s t-test. Differences were considered significant at P < 0.05 (*) and P < 0.01 (**). The line plots represent mean ± SD. (n = 4). (D) BubR1-dependent antiproliferation in HeLa cells with Cmpd-A treatment. Twenty-four hours after siNS or siBubR1 treatment, the cells were treated with Cmpd-A at the indicated concentration for 24 h. Relative ATP amounts were calculated based on chemiluminescence compared with the 0 nM chemiluminescence value in each siRNA treatment. Statistical analysis was performed using Student’s t-test. Differences were considered significant at P < 0.05 (*) and P < 0.01 (**). The line plots represent mean ± SD (n = 4).

To investigate the cellular antiproliferative activity of Cmpd-A, asynchronous HeLa cells were treated with a range of concentrations of Cmpd-A (0–1000 nM), and cell viability was determined based on the intracellular ATP concentration. As shown in Fig 3A, Cmpd-A potently suppressed the proliferation of HeLa cells in a concentration-dependent manner (GI_50_ = 80 nM) [29], and this antiproliferative effect was closely correlated with the percentage of pHH3-positive cells. These results indicate that, consistent with the molecular mechanisms of CENP-E, mitotic aberration appears to be involved in the antiproliferative mechanism of Cmpd-A and that pHH3 is a potential surrogate PD marker that can be used to monitor the cellular activity of Cmpd-A.

Immunofluorescence revealed that BubR1 was intensively localized at kinetochores of misaligned chromosomes in the Cmpd-A-treated cells (Fig 3B). Given that BubR1 leaves kinetochores when tension across sister chromatids is established (S1 Fig) [37], the SAC appears to be activated by BubR1 on the misaligned chromosomes in these cells. To clarify the correlation between the Cmpd-A-induced SAC activation and the Cmpd-A-induced antiproliferation, we determined caspase-3/7 activation as well as cell proliferative activity on the SAC-impaired condition induced by BubR1 knockdown. Twenty-four hours after siRNA treatment of non-silencing (siNS) or BubR1 (siBubR1), the siRNA-transfected cells were treated with Cmpd-A at the indicated concentration for 24 h. In response to Cmpd-A treatment, caspase-3/7 activation (Fig 3C, red line) and anti-proliferation (Fig 3D, red line) of the siNS-treated cells concurrently occurred in a dose-dependent manner. However, both caspase-3/7 and anti-proliferative activities were drastically reduced in the siBubR1-transfected cells (Fig 3C and 3D, blue lines), indicating that SAC activation predominantly contributes to Cmpd-A-induced apoptosis and antiproliferation if the SAC machinery is intact.

**Fig 4:**
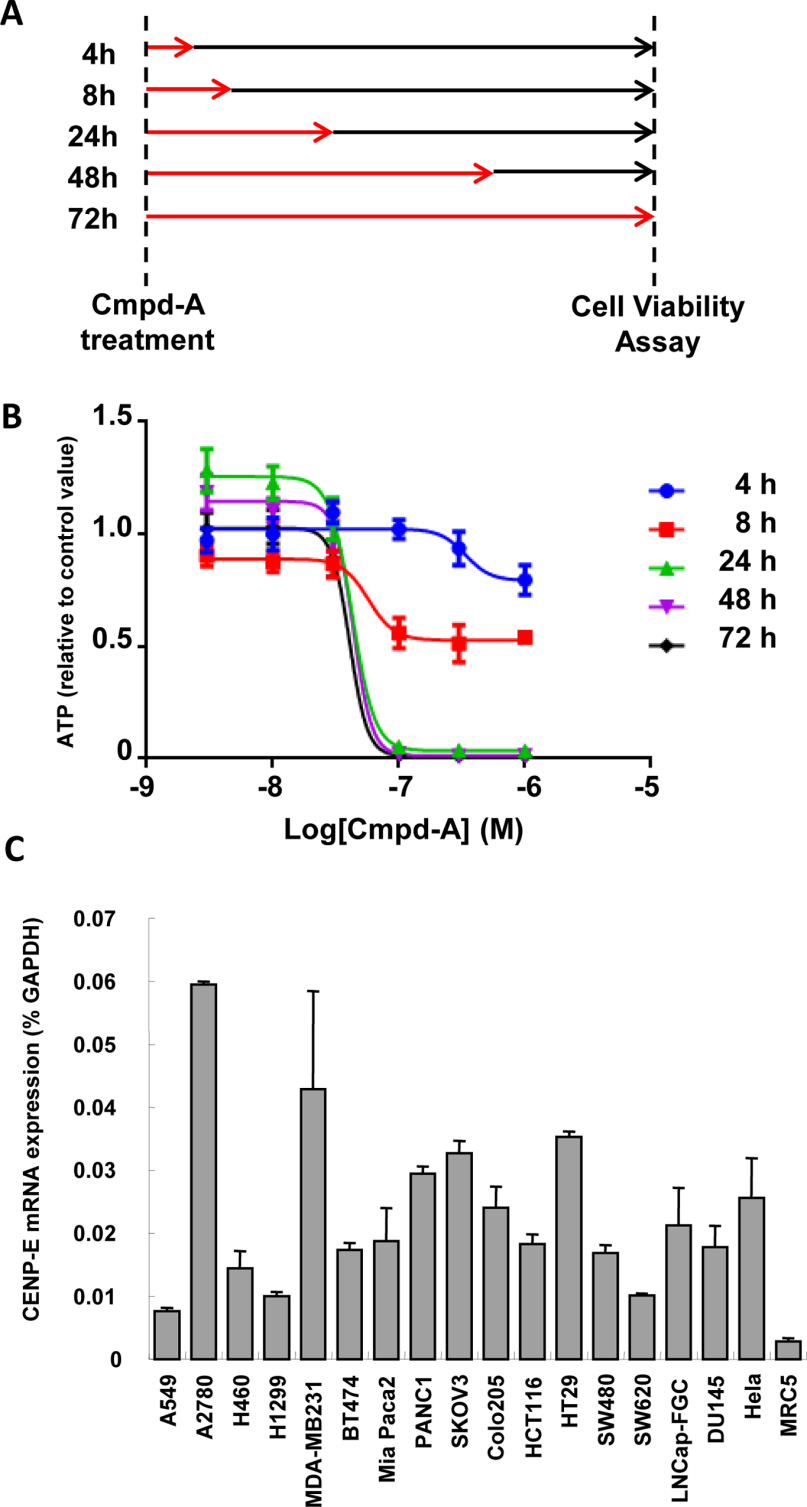
Time-dependent antiproliferative activity of Cmpd-A in HeLa cells. (A) Experimental schemes to asses time-dependent antiproliferative activity of Cmpd-A. HeLa cells were treated with Cmpd-A at the indicated concentrations for 4, 8, 24, 48, and 72 h (red arrows) and then the cells were cultured in Cmpd-A-free medium for 72 h (black arrows). Cells were collected 72 h after treatment for cell viability analysis. (B) Time-dependent antiproliferative activity of Cmpd-A in HeLa cells. The relative ATP concentration was calculated based on chemiluminescence compared with the 0 nM chemiluminescence value (control). Data are presented as mean ± standard deviation (n = 8). (C) Quantitative RT-PCR analysis of CENP-E in cancer cell lines and human skin fibroblasts (MRC5). CENP-E expression ratios were quantified using GAPDH expression in each cell line as a control. Data are presented as mean ± standard deviation (n = 3).

Next, the time required for exposure to Cmpd-A to induce potent antiproliferation was determined. HeLa cells were treated with Cmpd-A for 4, 8, 24, 48, or 72 h and then Cmpd-A was removed by changing the medium (Fig 4A). Cell viability assays were conducted 72 h after drug treatment commenced. Treatment with Cmpd-A for 24 h was as potent as the treatment for 48 or 72 h, whereas treatment for 8 h resulted in only mild antiproliferative activity (Fig 4B). These results demonstrate that continuous treatment with Cmpd-A is unnecessary, but intermittent treatment could be used to produce a potent antiproliferative effect.

### Cmpd-A exhibits antiproliferative activity in various cancer cell lines

**Fig 5:**
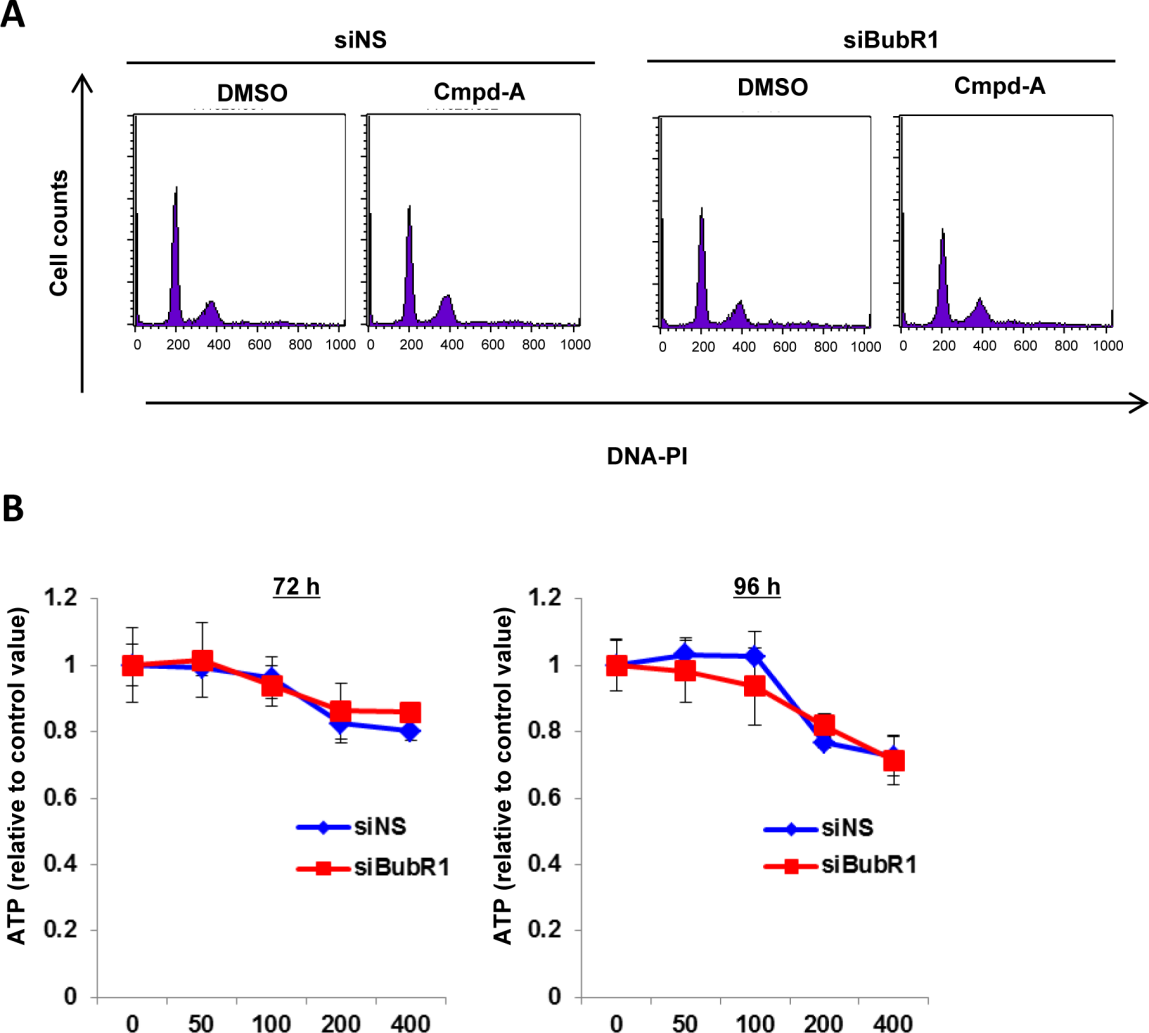
Effect of Cmpd-A on mitotic arrest and antiproliferation in untransformed skin fibroblast MRC5 cells. (A) Effect of Cmpd-A on mitotic arrest in siNS and siBubR1-transfected MRC5 cells. Twenty-four hours after siRNA transfection, MRC5 cells were treated with or without Cmpd-A (200 nM). Cells were collected 24 h after drug treatment for FACS assay. (B) Antiproliferative activity of Cmpd-A in siNS- (blue) and siBubR1-transfected (red) MRC5 cells. Twenty-four hours after siRNA transfection, MRC5 cells were treated with Cmpd-A at the indicated concentrations. Cells were collected 72 and 96 h after drug treatment for ATP assay. Relative ATP levels were calculated based on the chemiluminescence compared with the chemiluminescence value of 0 nM treatment for each. The line plots represent mean ± standard deviation (n = 3).

Because CENP-E mRNA expression is upregulated in a wide range of cancer cell lines compared with untransformed MRC5 cells (human skin fibroblasts; Fig 4C), we first assessed the antiproliferative activity of Cmpd-A in low CENP-E-expressing MRC5 cells. Cmpd-A treatment of MRC5 cells produced only a small response in terms of both mitotic arrest (Fig 5A, left panels) and antiproliferation (Fig 5B, blue lines). Unlike the results obtained in HeLa cells, siBubR1 caused little effect on cell cycle (Fig 5A, right panels) or antiproliferation (Fig 5B, red lines) in Cmpd-A-treated MRC5 cells, indicating that Cmpd-A does not modulate the SAC activity in MRC5 cells; thus, it exhibits little antiproliferative activity in this cell line.

**Fig 6:**
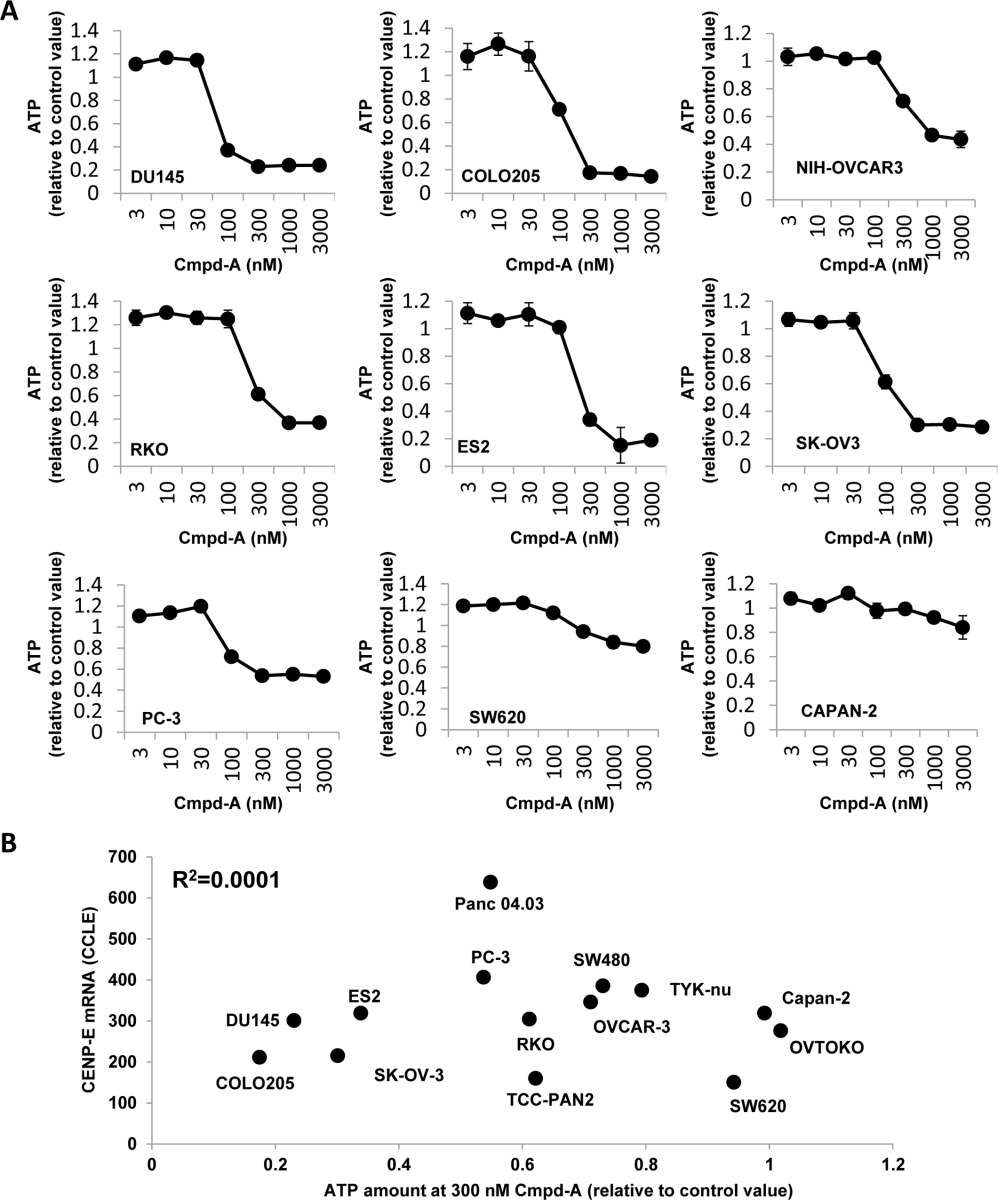
Cmpd-A exhibits potent antiproliferative activity in multiple cancer cell lines. (A) Antiproliferative activity of Cmpd-A in multiple cancer cell lines. DU145, COLO205, NIH-OVCAR3, RKO, ES2, SK-OV3, PC-3, SW620, and CAPAN-2 cell lines were treated with Cmpd-A for 3 days at the indicated concentrations. The relative ATP concentration was calculated based on the chemiluminescence compared with the 0 nM chemiluminescence value (control). Data are presented as mean ± standard deviation (n = 3). (B) Correlation between the antiproliferative activity of Cmpd-A and CENP-E mRNA expression in cancer cell lines. The X and Y axes indicate the relative ATP level at 300 nM Cmpd-A treatment and CENP-E mRNA levels in the 14 indicated cancer cell lines, respectively. The relative ATP concentration was calculated based on chemiluminescence compared with the 0 nM chemiluminescence value (control) in each cell line. The raw data of CENP-E mRNA expression was downloaded from the Cancer Cell Line Encyclopedia (http://www.broadinstitute.org/ccle/data/browseData) and processed with MAS 5.0 algorithm.

Next, the antiproliferative activity of Cmpd-A was investigated in multiple cancer cell lines. Cell viability assays with Cmpd-A were performed in 14 cancer cell lines: DU145, COLO205, NIH-OVCAR3, RKO, ES2, SK-OV3, PC-3, SW480, SW620, CAPAN-2, Panc 04.03, TCC-PAN2, TYK-nu, and OVTOKO (Fig 6A, S2 Fig, and S1 Table). Cmpd-A exhibited a clear antiproliferative effect in multiple cancer cell lines (Fig 6A). However, some cell lines, such as SW620 and CAPAN-2, were resistant to Cmpd-A and showed a similar response as MRC5 cells. Accordingly, we investigated the correlation between the sensitivity to Cmpd-A and CENP-E mRNA expression levels in cancer cell lines using the public gene expression database (Cancer Cell Line Encyclopedia; http://www.broadinstitute.org/ccle/data/browseData). Although MRC5 showed both low CENP-E expression and low sensitivity to Cmpd-A (Figs 4C and 5), the antiproliferative effects of Cmpd-A were not significantly correlated with CENP-E mRNA expression levels in tested cancer cell lines (R^2^ = 0.0001, Fig 6B). These results demonstrate that sensitivity of cancer cells to Cmpd-A cannot be predicted by their CENP-E expression alone, and other molecules or pathways may be responsible for the sensitivity of cancer cells to treatment with Cmpd-A. Taken together, CENP-E appears to be a critical gene for proliferation in multiple cancer cells, and the CENP-E inhibitor Cmpd-A could exhibit antiproliferative activity in a broad spectrum of cancers.

### PK, PD, and antitumor efficacy of Cmpd-A in a COLO205 xenograft nude mouse model

**Fig 7:**
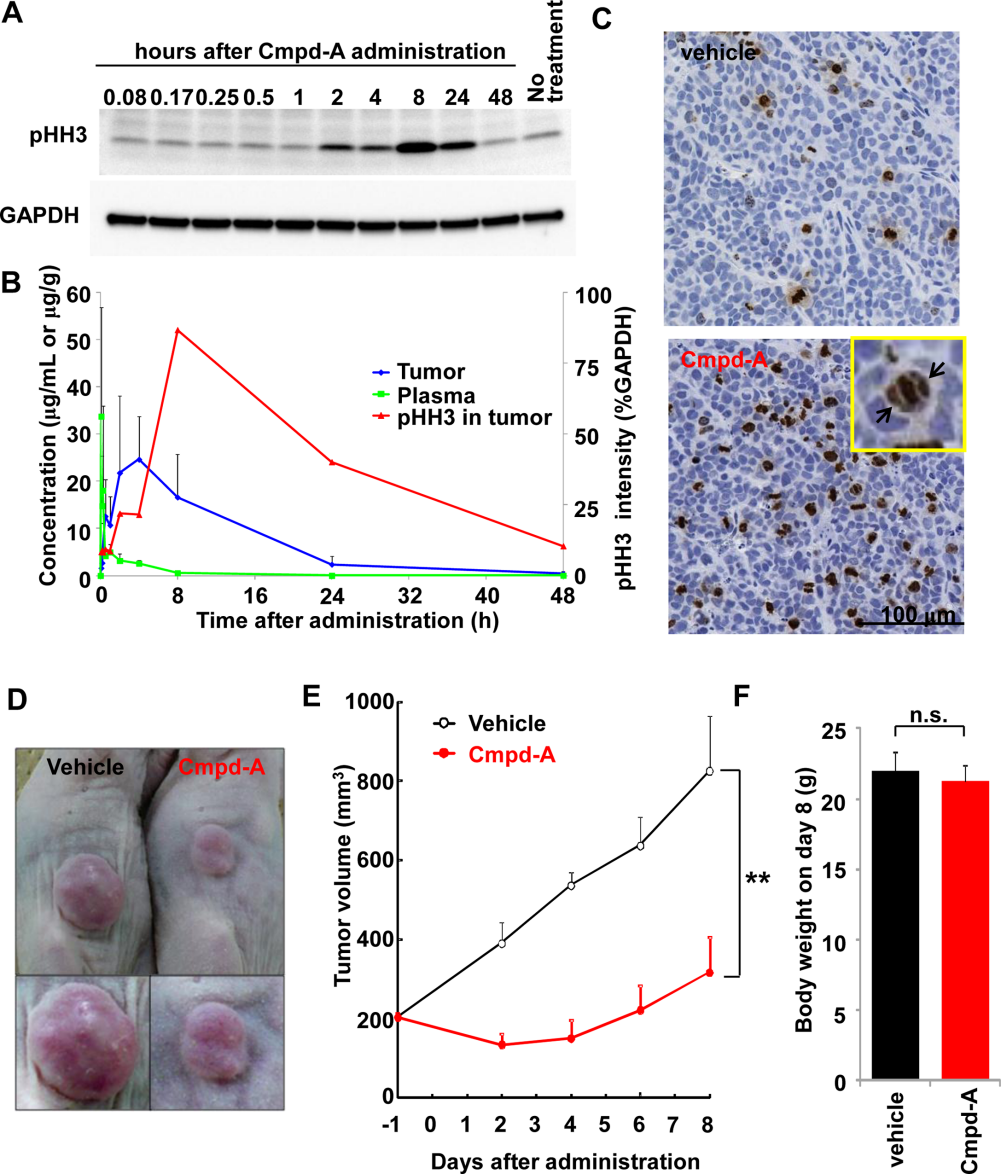
PK/PD and antitumor efficacy of Cmpd-A in a COLO205 xenograft nude mouse model. (A) Expression of pHH3 in COLO205 xenografts at the indicated time points after the intraperitoneal administration of Cmpd-A at a dose of 100 mg/kg. (B) Time-dependent PK and PD of Cmpd-A. The green, blue, and red lines indicate plasma concentration, tumor concentration, and tumor pHH3 intensity, respectively. The pHH3 intensity was quantified using the results shown in (A). (C) Immunohistochemistry of pHH3 in the tumor sections from COLO205 xenograft nude mice 24 h after the intraperitoneal administration of Cmpd-A at 100 mg/kg. Black arrows indicate misaligned chromosomes in sections from COLO205 xenografts treated with Cmpd-A. (D) Antitumor efficacy of Cmpd-A in the COLO205 xenograft nude mouse model. COLO205 xenografted nude mice intraperitoneally injected with Cmpd-A at 100 mg/kg or vehicle three times (at 0, 8, and 24 h) on the first day of the study. Representative tumors 8 days after the administration of vehicle or Cmpd-A are shown. (E) Efficacy data plotted as the mean tumor volume (mm^3^ ± standard error of the mean; n = 5) in COLO205 xenograft nude mice treated with Cmpd-A (red) or vehicle (black). Statistical analysis was performed using Student’s t-test. Differences were considered significant at P ≤ 0.05 (*) and P ≤ 0.01 (**). (F) Bodyweight comparison of COLO205 xenograft nude mice 8 days after the administration of Cmpd-A (red) or vehicle (black). Data are presented as mean ± standard deviation (n = 5). Statistical analysis was performed using Student’s t-test. Differences were considered significant at P ≤ 0.05 (*) and P ≤ 0.01 (**).

The PK and PD of Cmpd-A were investigated in a xenograft nude mouse model. COLO205 cells were selected for this *in vivo* study because this cell line was sensitive to Cmpd-A in the *in vitro* study (Fig 6). Furthermore, the COLO205 model is well established because of its usability, relatively high take rates, and growth rates [24]. COLO205 xenografted mice were treated with a single intraperitoneal injection of Cmpd-A at 100 mg/kg. Tumor and plasma samples were collected at the indicated time points, and the concentrations of Cmpd-A (PK) and the pHH3 level in tumors (PD) were measured (Fig 7A and 7B). The plasma concentration of Cmpd-A peaked at 33.7 μg/mL 5 min after administration and then rapidly decreased (Fig 7B green line). A delayed peak concentration of 24.6 μg/mL was observed in the tumor samples 4 h after administration (Fig 7B blue line). The peak level of pHH3 in the tumor samples was further delayed by several hours from the peak concentration of Cmpd-A in the tumor samples. The level of pHH3 increased from 2 h to a peak at 8 h after administration, then decreased to approximately half that level by 24 h and was minimal at 48 h (Fig 7B red line). Accordingly, immunohistochemistry of the tumor sections also showed the accumulation of pHH3-positive cells (Fig 7C lower panel) as well as misaligned chromosomes (black arrows) 24 h after the administration of Cmpd-A. These results demonstrate that although potent effects related to PD are evident 8 h after the administration of Cmpd-A, the effects are not sustained for more than 24 h after administration.

The *in vitro* washout experiments revealed that an 8-h exposure is not long enough to induce potent growth inhibition, whereas a 24-h exposure exhibited a similar antiproliferative activity as the 72-h exposure (Fig 4B). Therefore, the PD effects sustained for 24 h were expected to induce antitumor activity in the xenograft mouse model. However, the PK/PD time-course data for the intraperitoneal administration of Cmpd-A indicated that the PD effects in the COLO205 xenograft nude mouse model decreased between 8 and 24 h after drug administration. To sustain the intensive PD effect for ~24 h *in vivo*, Cmpd-A was intraperitoneally administered to the COLO205 xenograft nude mouse model at a dose of 100 mg/kg three times (0, 8, and 24 h) on the first day of the efficacy study. As shown in Fig 7D and 7E, the administration of Cmpd-A on this schedule significantly enhanced antitumor efficacy in the COLO205 model. The antitumor activity (T/C %) was 11% on Day 8 (p < 0.01), but there was no significant bodyweight loss compared with control COLO205 xenograft nude mice treated with vehicle. The efficacy study was completed on Day 8 because tumors in the control mice were starting to rupture (Fig 7D, left). Taken together, these results demonstrate that the administration of Cmpd-A causes mitotic aberration and activates the SAC machinery in COLO205 xenograft nude mouse models and exhibits significant antitumor activity.

## Discussion

A time-dependent CENP-E inhibitor, Cmpd-A, was developed based on the biochemical screening of the ATPase activity of the CENP-E motor domain [29]. The present study investigated the enzymatic mode of action, cellular morphology, antiproliferative cellular activity, PK and PD, and *in vivo* antitumor activity of Cmpd-A. The results demonstrate that Cmpd-A potently suppresses cellular proliferation in multiple cancer cell lines and exhibits antitumor activity in the COLO205 xenograft nude mouse model. These findings suggest that small molecules targeting the CENP-E motor domain are potential targets for anticancer drugs. However, considering how difficult it is to achieve chemical optimization with adequate pharmaceutical potency, to date, only one CENP-E small- molecule inhibitor, GSK923295, has been evaluated in Phase I clinical trials [24, 26]. Peripheral neuropathy, one of the major adverse effects of tubulin binders such as taxanes or vinca alkaloids [2], was not evident in the clinical trial of GSK923295 [26]. The results of the present study demonstrate that the use of CENP-E inhibitors as anticancer drugs could potentially avoid peripheral neuropathy associated with tubulin-binding chemotherapeutic agents, presumably because CENP-E inhibitors affect proliferating cells but not non-proliferating peripheral neuronal cells.

Cmpd-A is a time-dependent CENP-E inhibitor with an ATP-competitive-like behavior (Fig 1A), whereas GSK923295 is an ATP-uncompetitive CENP-E inhibitor [24]. The cellular concentration of ATP is relatively high (1–10 mM) [38]. Therefore, ATP-competitive inhibitors, which overcome high concentrations of cellular ATP, are expected to have a lower chance of either reaching the ATP-binding pocket of the target molecules or staying bound to their target long enough for effective inhibition. In general, ATP-competitive inhibitors tend to impair cellular activities rather than the enzymatic activities of cell-free systems. However, Cmpd-A with an ATP competitive-like behavior is a time-dependent CENP-E inhibitor. This time-dependent behavior of Cmpd-A might result in the conformational change of the ATP binding site of CENP-E when it binds to the L5 allosteric site [32, 33]. Because of this unique enzymatic mechanism, Cmpd-A is expected to inhibit CENP-E motor activity effectively even at high cellular ATP concentrations. This action results in potent cellular activity and potential growth inhibition of cancer cells (GI_50_ = 80 nM in HeLa cells [29]).

Although it is difficult to directly monitor CENP-E cellular activity modulated by Cmpd-A, the inhibitory effects of Cmpd-A were determined in cells with pole-proximal misaligned chromosomes, in which the inter-kinetochore tension was significantly weakened. In parallel with the mitotic aberration induced by Cmpd-A, the mitotic marker pHH3 was also elevated *in vitro* and *in vivo*. Furthermore, pHH3 levels and the antiproliferative activity of Cmpd-A were correlated. Although pHH3 is not a biomarker to monitor CENP-E motor activity directly, this correlation indicates that pHH3 could potentially be used as a surrogate for the PD biomarker as well as an efficacy marker. Further preclinical studies in multiple tumor models are needed to develop the utility of pHH3 as a biomarker for CENP-E inhibitors.

Although the antitumor activity of GSK923295 is being evaluated clinically, preclinical studies have revealed that CENP-E mRNA expression does not correlate with sensitivity to the CENP-E inhibitor in cancer cells (Fig 6B and [24]), suggesting that CENP-E expression is not a feasible biomarker for predicting tumors that are sensitive to the CENP-E inhibitor. Thus, identification of a novel patient selection biomarker(s) will have a major impact on clinical strategies. The preclinical studies of GSK923295 have indicated that neuroblastoma and pediatric cancers might be potential target indicators for CENP-E inhibitors [24, 27, 28]. More recently, a potent and selective CENP-E inhibitor, PF-2771, was reported as the second CENP-E small -molecule inhibitor to exhibit antitumor activity in preclinical animal models [39]. These preclinical studies revealed that basal-like breast cancer cell lines are sensitive to PF-2771. Therefore, CENP-E inhibitors may also be effective for patients with triple-negative/basal-like breast cancer. Furthermore, our preclinical studies revealed that under SAC-attenuated conditions, Cmpd-A induces unequal chromosome segregation into daughter cells via mitotic slippage, resulting in aneuploid-associated apoptosis in a p53-dependent manner [29]. Therefore, p53 could be a potential predictive biomarker for CENP-E inhibitors if cancers spontaneously exhibit attenuation of the SAC machinery. Further investigations of the molecular mechanisms of CENP-E inhibitors will provide an important insight into target indicators and optimal clinical strategies.

## Conclusions

We investigated the enzymatic mode of action of the novel time-dependent CENP-E inhibitor Cmpd-A and its effects on cellular morphology. Cmpd-A exhibited potent antitumor activity in a COLO205 xenograft nude mouse model and antiproliferative activity in multiple cancer cell lines. These findings suggest that, in addition to other non-microtubule targeting drugs, small molecules targeting the CENP-E motor activity represent the next generation of mitotic inhibitors and have important potential as anticancer drugs.

## Supporting Information

S1 Fig BubR1 localization at prometaphase and metaphase in HeLa cells. Representative immunofluorescence of BubR1 in HeLa cells at prometaphase (upper panels) and metaphase (lower panels) without Cmpd-A treatment. Green, red, and blue signals indicate BubR1, CENP-B, and DAPI-stained DNA, respectively. White bar indicates 10 μm.
(TIF)

S2 Fig Anti-proliferative effects of Cmpd-A in multiple cancer cell lines. TYK-nu, OVTOKO, Panc04.03, TCC-PAN2, and SW480 cell lines were treated with Cmpd-A for 3 days at the indicated concentrations. The relative ATP concentration was calculated based on the chemiluminescence compared with the 0 nM chemiluminescence value (control). Data are presented as mean ± standard deviation (n = 3).
(TIF)

S1 Table Relative Growth Values in Various Cancer Cell Lines with Cmpd-A Treatment (TIF)
