# Supplementary figures and images for: A Novel Time-Dependent CENP-E Inhibitor with Potent Antitumor Activity
Source: PLoS One. 2015 Dec 9;10(12):e0144675. doi: 10.1371/journal.pone.0144675 (PMC4674098; doi:10.1371/journal.pone.0144675)

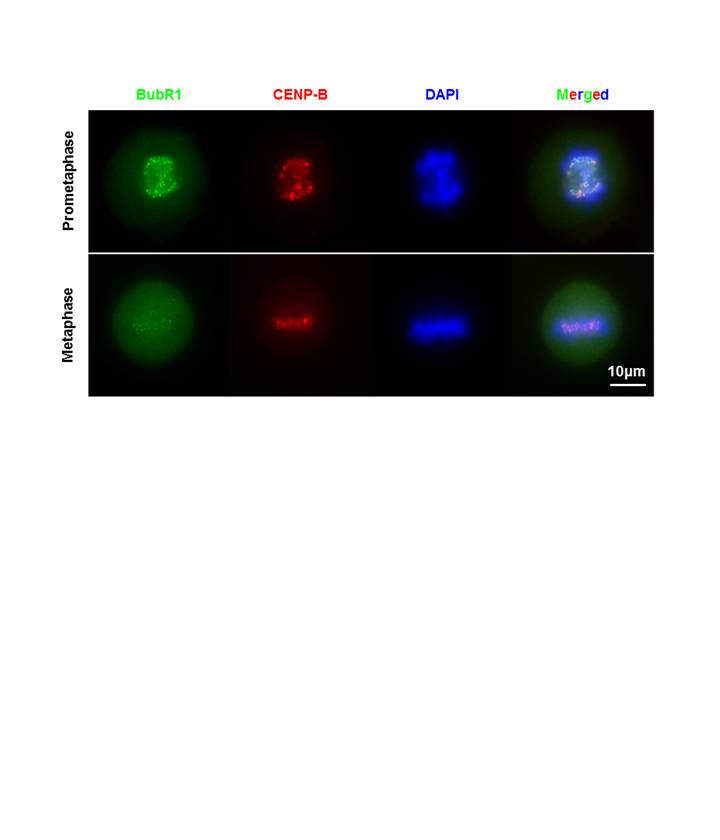

Supplement: S1 Fig — Representative immunofluorescence of BubR1 in HeLa cells at prometaphase (upper panels) and metaphase (lower panels) without Cmpd-A treatment. Green, red, and blue signals indicate BubR1, CENP-B, and DAPI-stained DNA, respectively. White bar indicates 10 μm. (TIF) [file pone.0144675.s001.TIF]

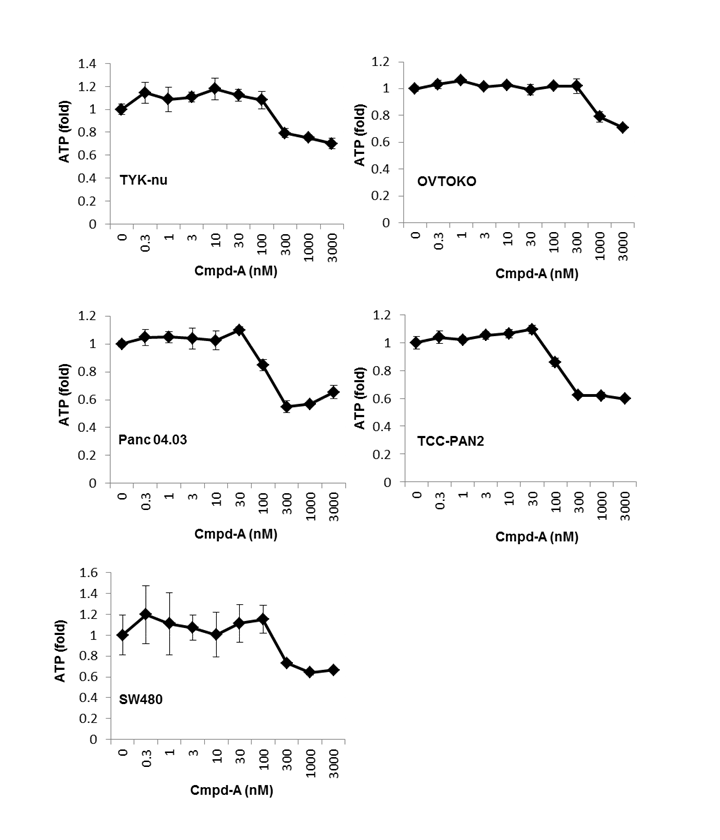

Supplement: S2 Fig — TYK-nu, OVTOKO, Panc04.03, TCC-PAN2, and SW480 cell lines were treated with Cmpd-A for 3 days at the indicated concentrations. The relative ATP concentration was calculated based on the chemiluminescence compared with the 0 nM chemiluminescence value (control). Data are presented as mean ± standard deviation (n = 3). (TIF) [file pone.0144675.s002.TIF]

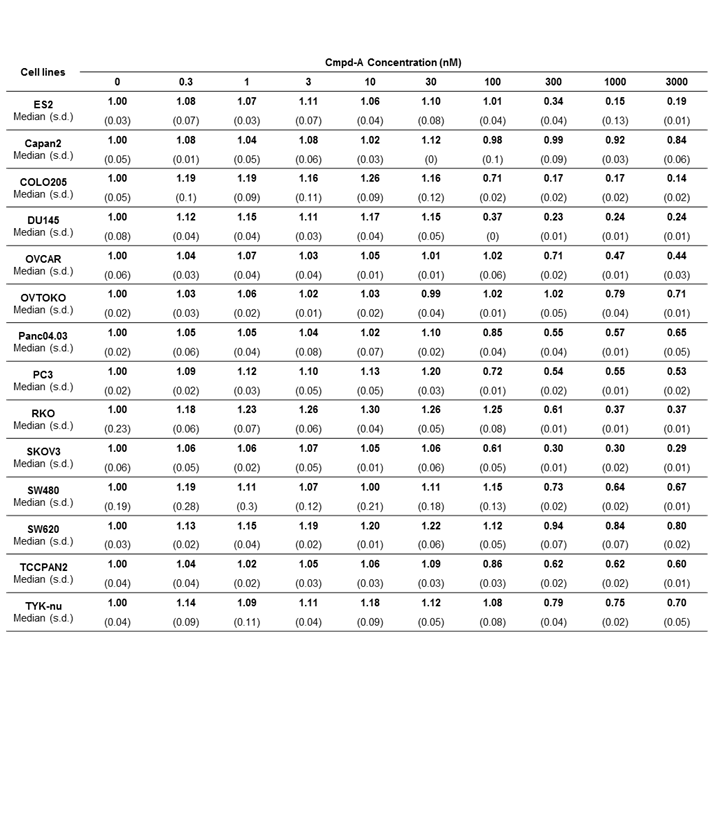

Supplement: S1 Table — (TIF) [file pone.0144675.s003.TIF]
